# Regulation of community advisory boards during conduct of clinical trials in Uganda: a qualitative study involving stakeholders

**DOI:** 10.1186/s12913-023-09136-w

**Published:** 2023-02-06

**Authors:** Andrew Ojok Mijumbi, Levicatus Mugenyi, Mastula Nanfuka, Collins Agaba, Joseph Ochieng

**Affiliations:** 1 The Aids Support Organization, Kampala, Uganda; 2 Makerere University Lung Institute, Kampala, Uganda; 3 Makerere University Johns Hopkins University Collaboration, Kampala, Uganda; 4 CORE Consulting Limited, Kampala, Uganda; 5 Department of Anatomy, School of Biomedical Sciences, College of Health Sciences, Makerere University, P.O Box 7072, Kampala, Uganda

**Keywords:** Regulation, Community advisory boards, Clinical trials, Stakeholders Uganda

## Abstract

**Background:**

Community advisory structures such as Community Advisory Boards (CABs) play an important role of helping researchers to better understand the community at each phase of the clinical trial. CABs can be a source of accurate information on the community, its perception of proposed research and may identify factors that make community members vulnerable to the problem under investigation. Although CABs help to build mutually beneficial relationships between the researcher(s) and the communities in which the clinical trial is being implemented, effective engagement would require ethical guidance and regulatory oversight.

The study assessed the stakeholders’ perspectives regarding the regulatory oversight of CABs in Uganda.

**Methods:**

This was an exploratory study employing qualitative methods of data collection and analysis. Key informant interviews (KIIs) with the trial investigators, CAB chairpersons, community liaison officers, regulators and Research Ethics Committee (REC) chairpersons were conducted. A KII guide was designed and utilized during key informant interviews. The guide included questions on role of investigators and CAB members in clinical trials; challenges of community engagement; facilitation of CABs; regulatory oversight of CABs; work relationships between investigators and CABs; and opinions on how community trials should be conducted among others. All interviews were conducted in English.

Qualitative data were transcribed verbatim. A code book was generated based on the transcripts and study objectives. Thematic analysis was used to analyze data and identify themes. Atlas ti was used to support data analysis.

**Results:**

Of the 34 respondents, 35.3% were investigators, 32.3% CAB chairpersons, 23.5% research regulators/REC Chairs and 8.8% community liaison officers. The findings of the study revealed that CABs are appointed by the research institution/researcher, operate under the guidance of the researcher with limited independence. Additionally, the CABs provide voluntary service and lack guidelines or regulatory oversight. Four themes emerged.

**Conclusion:**

The operations and activities of CABs are not regulated by the national regulators or RECs. The regulatory oversight of CABs should be based on contextualized ethical guidelines. Need for additional training in research ethics, community engagement and sensitization on available ethics guidelines for research.

**Supplementary Information:**

The online version contains supplementary material available at 10.1186/s12913-023-09136-w.

## Introduction

Community engagement has been a focus for global debate regarding ethical conduct of research. Many national and international guidelines recommend engagement of research communities in a sustained manner from the onset, design, conduct, conclusion and dissemination of research results, to enhance the quality and outcomes of research, and for the protection of communities [1–4].

Such engagement is expected to address the ethical requirement that research be responsive to the health needs of the community, minimize potential exploitation and abuse of research communities as well as facilitate uptake of research results [5]. Effective community engagement requires contextualized ethical guidelines that facilitate regulatory oversight and ensure community protections [6, 7]. Since well-regulated engagement is expected to minimize aspects like researchers limiting engagement to institutional gatekeepers [8].

Community advisory structures play an important role in linking the researcher to the study communities and can provide input into informed consent, educational materials to be used in the research, input on issues of cultural sensitivity and appropriateness of incentives given in the research [1, 2]. Community advisory structures such as Community Advisory Boards (CABs) play an important role of helping researchers to better understand the community at each phase of the clinical trial [3]. In the pre- trial phase CABs are a source of accurate information on the community, its perception of the proposed research and may identify factors that make community members vulnerable to the problem under investigation. In the protocol development phase they provide, inputs for the design of appropriate recruitment and retention strategies, aid in translation of study materials into local languages and the development of a glossary of research terms. In the implementation phase, they help to channel accurate information to the community and educate it about research, while bringing community needs and concerns to researchers. In this way, CABs provide bi-directional feedback between researchers and the community. Thus, CABs help to build mutually beneficial relationships between the researcher(s) and the communities in which the clinical trial is being implemented [9].

Although CABs are considered important during conduct of clinical trials, their formation and ethical roles have been studied with equal praise and critique [10–16]. Challenges include poor CAB management, lack of formal participation structures, language barriers, low literacy levels, lack of commitment, budgets, and operational challenges [17–19]. Other issues that affected the effectiveness of CABs in clinical trials have been documented as limitations of guidelines for their operations [20].

Yet there is need to address the long-standing questions and gaps around strengthening, implementation and evaluation of stakeholder engagement in global health and clinical trials [21–27]. Despite the importance of CABs during the conduct of clinical trials, and their requirement by the national guidelines and the national regulatory agency, there is a paucity of evidence on the regulation and functionality of CABs in Africa, Uganda inclusive [28–30]. A recent systematic review of community engagement strategies in biomedical research in Africa found only eight articles that focused on the role and functions of CABs [31]. Moreover, most of the available studies have been done on individual or study specific CABs [32]. Only one study was identified to have investigated membership practices, selection criteria, qualifications, and challenges of CABs at six Ugandan HIV clinical research institutions [9]. Yet CAB members were observed not able to critically evaluate studies for the presence of exploitative elements in Bangkok, Thailand [16]. Additionally, despite the requirement for CABs during conduct of clinical trials by the national guidelines, there is no mechanism for providing regulatory oversight on how such CABs operate in Uganda. And since CAB recruitment and supervision has been left to the researcher with inadequate guidance and no oversight by the research regulatory agencies, questions about their functionality and usefulness keep arising.

The study assessed the stakeholders’ perspectives regarding the regulatory oversight of CABs in Uganda.

## Methods

### Design and setting

This was an exploratory study employing qualitative methods of data collection and analysis. The study was conducted in Uganda between March and October 2020 covering 19 research institutions that were conducting clinical trials according to the National Drug Authority database(33). We randomly selected 26 clinical trials out of the 74 that existed in the database including both completed (not more than 1 year) and ongoing trials. We randomly selected 1 clinical trial per institution conducting 1 to 4 trials, 2 clinical trials per institution conducting 5–9 trials, 3 clinical trials from each institution conducting 10–14 trials, and 4 clinical trials from institutions conducting 15 or more trials. This resulted into a total of 26 clinical trials from the 19 research institutions. All CABs attached to the selected clinical trials were included in the study.

#### Participants

The study recruited 34 respondents purposively selected including 11 CAB chairpersons, 12 trial investigators, and 3 community liaison officers. A community liaison officer is a person based at the research institution and his/her role is to link the research institution or investigators to the communities where they wish to conduct the study. In addition, research regulators 1 from the National Drug Authority, 1 from the Uganda National Council for Science and Technology (UNCST) and 1 from the Uganda National Health Research Organization at the national level as well as 5 chairpersons of REC were purposively selected to participate as key informants. The investigators of this study had no prior working relationships relationship with most of the participants.

### Data collection

Data were collected by a team of individuals with expertise and experiences in qualitative research methods. Data collection led mainly by a male MD with bioethics expertise and an academician, a male behavioral scientist with extensive experience in qualitative research, and a male social scientist with experience in qualitative data collection. They were assisted by a male and female masters level social scientists who are qualitative researchers with extensive experience. Key informant interviews (KIIs) lasting 45–60 minutes were held with all targeted interviewees. Face to face Interviews were conducted in private locations identified by participants at their respective work places. The KII were guided by a semi-structured interview guide that was developed for this study and pretested before use (submitted as Additional materials file). We considered a purposive sample of individuals who had a good understanding of community engagement in clinical trials in Uganda. Issues discussed included role of investigators and CAB members in clinical trials; facilitation for CABs; and regulatory oversight of CABs. All interviews were conducted in English and audio recorded, supplemented by notes taken by the assistants.

### Data analysis

Qualitative data were transcribed verbatim. A code book was generated based on the transcripts and study objectives. The codes were later discussed and agreed upon. The coding was done both deductively based on the study predetermined themes and, inductively to identify emerging themes. Transcripts were further reviewed for emerging themes which were integrated into the thematic matrix. The researcher was involved in applying and confirming application of codes across all transcripts and disagreements were resolved by cross checking with the recorded data. Thematic content analysis was used to analyze qualitative data. Atlas ti was used to support data analysis. Qualitative information is presented as narratives and quotes.

#### Ethical considerations

Ethical review and approval was obtained from the TASO REC (TASOREC/065/2019-UG-REC-009) before study implementation. Registration and clearance of the protocol was done by UNCST (SS 5120). The research team sought administrative clearance from institutions and community gatekeepers. All participants were adults of 18 years and above and provided written informed consent before enrolment into the study. All the methods were carried out in accordance with relevant national and international guidelines and regulations. No participant identifying information was recorded.

## Results

Of the 34 respondents, 29.4% were investigators, 29.4% CAB chairpersons, 29.4% research regulators/REC Chairs and 11.8% community liaison officers. Fifty-nine percent were male and had varying occupations and demographic characteristics Table 1.

**Table 1:** Demographic characteristics of key informants

Characteristics	
**Investigators**	***N*** **= 10**
**Sex, n (%)**	
Male	7 (70%)
Female	3 (30%)
Age in years, median (Range)	40 (31–62)
Area of specialization, n (%)	
Epidemiologist	3 (30%)
Public health	3(30%)
Pediatrician	2 (20%)
Medical Doctor	2 (20%)
**CAB chairpersons**	***N*** **= 10**
**Sex, n (%)**	
Male	5 (50%)
Female	5 (50%)
Age in years, median (range)	48 (32–75)
Area of specialization, n (%)	
Epidemiologist	2 (20%)
Medical Doctor	1 (10%)
Social worker	2 (20%)
Teacher	2 (20%)
Theology	2 (20%)
Civil servant	1 (10%)
**REC chairpersons**	***N*** **= 7**
**Sex, n (%)**	
Male	5 (71.4%)
Female	2 (28.6%)
**Age in years, median (range)** ***declined***	
**Area of specialization, n (%)**	
Epidemiologist	1 (14.3%)
Sociologist	1 (14.3%)
Pediatrician	1 (14.3%)
Physician	2 (28.6%)
Pharmacist	1 (14.3%)
Medical Doctor	1 (14.3%)
**Community Liaison Officers**	***N*** **= 4**
**Sex, n (%)**	
Male	2 (50%)
Female	2 (50%)
Age in years	
Median (Range)	44 (28–54)
Area of specialization, n (%)	
Sociologist	1 (25%)
Public health	1 (25%)
Clinical epidemiologist	1 (25%)
Teacher	1 (25%)
**Regulators**	***N*** **= 3**
**Sex, n (%)**	
Male	1 (33.3%)
Female	2 (66.7%)

The main findings of the study include the fact that CABs are recruited and work for the researcher, their operations are not regulated and no ethical guidelines to inform their practice Table 2.

**Table 2:** Main findings

Number	Main findings	Themes
1	CABs are appointed by the research institution/researcher	Current practices for CAB establishment and monitoring
2	Operate under the guidance of the researcher with limited independence	Need for regulatory oversight
3	Operations are not standardized	Appropriate training and skills
4	Lack guidelines or regulatory oversight by the UNCST and or RECs	Challenges faced by CABs
5	Operations and activities need regulation by the national regulators or RECs	
6	Voluntary nature of CAB services	

### Current practices for establishment and monitoring of CABs activities

The formation of CABs takes on a consultative process among the different stakeholders together with the target trial community. Research institutions utilize different approaches in the process of identification and selection of CAB members. For most research institutions, the Principal Investigators and the research team write to organizations or different constituencies relevant in the implementation of a proposed trial. Thus, the community sits and selects the person they want to represent them.

*“It is the communities to identify people. It is the community that selects representatives because for you as the researcher, how would you know who to bring on board? You don’t know but you approach structures within the community and request them to identify people who can sit on the board namely religious bodies, local leaders, HIV groups, groups out there working with HIV, and the media. We approach them and ask them to identify a person to represent them on the board.”* ***KII, Investigator KII 004.***

*“A CAB member is nominated from the community as somebody with interests of the community and they are from that locality or the district. And preferably people who have some experience in public health.”* ***P027 CAB Chair.***

It was noted that in some cases, investigators would directly identify individuals or experts relevant to the conduct of their clinical trial and write to them asking them to be part of their CAB or identify the potential members.

*“It is a process that requires time and resources. We have formed ours. You meet as a team to set it up. You identify the different areas that the team feels are key to their project. We write letters to the head of those constituencies. If it is the teachers, you write to the teachers’ association and if it is the VHTs, the leader of VHTs. We give all the constituencies time to identify their persons and respond. So, when they do, we also wait for all the other identified constituencies to second their representatives. Thereafter, we train, orient them and deploy.”* ***KII, Investigator*** *KII 005.*

Our findings also revealed that in other instances, research institutions team up together and agree to form a joint CAB that can serve them across the different clinical trials they are undertaking.

*“We met as four partners and identified groups of interest to us. For example, in one trial, we are working in schools and therefore we needed school representations. Being that it is health we needed a health expert representation and we all agreed as partners that we shall need representation from DHO’s office, teachers, religious and cultural leaders. Then, for HIV vaccine trials, we needed expert clients.”* ***KII, Investigator KII 005.***

The process of identification and formation of the CAB requires time and financial resources. More so, investigators reported that some trials do not provide budget lines for CAB activities. And if they do, the resource envelop is so small.

*“Each institution has its own way of selecting community advisory boards. But the cardinal principle is that the community advisory board should be independent of the research institution and their role should be more inclined to being advisors and not being part of the research team. We know the dominant faiths in Uganda. In such a case, you would need to go to the religious denominations and request them to nominate someone to represent their constituency on our CAB.”* ***KII, Community Liaison Officer KII 007.***

Our findings further revealed that whereas there is guidance on formation and selection of members of the CAB, there were variations in terms of the number of members serving on a CAB and the tenure of office a member is supposed to serve. The expectation is that the number of members serving on all CABs and their tenure of office should be uniform across the board. However, this was not the case. For example, when asked how long they are supposed to serve on a CAB, CAB chairs’ responses ranged from 1 to 15 years with a median duration of 5 years (interquartile range (IQR): 3–7).

*“For the tenure of office, we have a very big problem because it has been very vague. We have not had rules that you are supposed to serve for three years or one year and then another team takes over. For the CAB, how we have been doing it is we constitute one for a particular study and it serves the entire period of the study. But there are times where studies would overlap and the same CAB was serving more than one study. Except what would change will be like the community representative because the study needs to have study participants coming in but the same CAB would remain.”* ***KII, Investigator KII 025.***

*“For our institutional CAB, each person is supposed to serve a term of two years renewable once. So, at the end of the four years you are replaced and this is done in consultation with the seconding constituency. Based on the individual performance, they will recommend the person to have another term of office. But also, we evaluate the performance of that particular person by looking at how this person’s attitude has been throughout.”* ***KII, Community Liaison Officer KII 007.***

It was noted that in the current national research ethics guidelines, CABs are supposed to be formulated by the Principal Investigators for the concerned clinical trials. However, some of the key informants revealed that there were other considerations for the selection of an individual to serve on a CAB. Apart from the language and cultural values, some CAB members are selected because they are influential in their communities they come from such as the teachers, religious leaders, healthcare providers, and other technocrats.

*“Then we also have a section on Community Advisory Boards whereby it is required for a researcher to set up a CAB if they are going to do a clinical trial and I have actually seen it mostly in clinical trials. Then within the proposal, we look out during the review to have a community engagement plan. So, protocols and proposals are not approved when there is no community engagement plan and currently within the national research management system the online research application platform, we have embedded the requirement of a community engagement plan. So, it is going to be a must,*.” ***KII, Research regulator KII 037.***

*“The local language is part of the culture but in a way, a CAB member may not have the knowledge of the local language. For example, if you are dealing with teachers in a school, you may have this particular influential teacher in the village who does not speak the local language but the community respects them.”* ***KII, Investigator*** *KII 005.*

#### Mechanisms for monitoring CAB activities

Respondents noted that the Principal Investigators regulate CAB activities. In some cases, it was reported that CABs regulate themselves with guidance from community outreach teams since their work is voluntary.

“*… they are actually supposed to regulate themselves but with support from the community team because if you woke up today and recommended me for a CAB, I would ask you, “What are we doing here? So, they are guided by the community outreach team but they should be able to work independently.”* ***KII with Investigator KII 012.***

*“In my opinion I do not think that we should be first of all regulating them against what? What is it that they can do wrong these CABs because they should be able to regulate where we think people might be wrong?”* ***KII with REC chair KII 034.***

*“It is one of the gaps and I have always mentioned it, we need an M&E framework to monitor what the CAB members are doing.”* ***P021 CAB Chair.***

According to study findings investigators recommended regulators to determine the kind of guidelines or manuals to standardize operations of CAB activities.

*“I think that would depend on the regulators to determine whether they want to regulate CABs or not? And if they decide to regulate, they need to ensure they standardize the way CABs work.”* ***KII with Investigator KII 004.***

Respondents observed that since the REC has been mandated by the UNCST to review protocols, monitor implementation of research activities and ensure dissemination, the RECs can as well monitor the CABs. Other regulators proposed that investigators should do some monitoring of CABs through mutual discussion of expectations by both parties.

*“RECs review the protocol process and follow up implementation of research. So, the RECs can have some guidelines on monitoring CABs and also make reports on their failures and achievements.”* **KII Regulator KII 035.**

### Need for regulatory oversight

Findings revealed that the ultimate purpose of regulatory bodies is protection of the rights and safety of the research participants as well as ensuring good quality scientific research studies. Regarding safety of participants, regulatory bodies try to ensure that researchers do not harm or exploit study participants. Regulators further noted that their role was to ensure that the needs of the community are taken into consideration and that researchers pay attention to their conduct in the community by following the Standard Operating Procedures (SOPs).

#### Setting a regulatory framework for CAB

Several suggestions were given for regulation of CAB activities and these ranged from having a specific CAB for each clinical trial, conducting a Needs Assessment of CABs intended purpose, roles, challenges and having an individual at UNCST to oversee CAB activities. Regulators recommended that each trial have its own CAB rather than having one CAB for all the trials at a given research institution like it is for most of the institutions in Uganda.

*“… it would be good to have different CABs for different clinical trials than having the research institute set up one CAB because each trial has different demands and also has different scientific elements, so it has a risk of someone having sort of disinformation.” KII* ***Regulator KII 037.***

*“The structure, the researcher can propose within their proposal and then submit names and then where they are coming from and then the UNCST would approve. So, it comes from the researcher’s proposal then to UNCST then UNCST will be like okay, this CAB for this particular research, the people are from this particular area and are well conversant and are not so busy. They are not like in 5 trials to give this particular trial all the time it requires.” KII* ***Regulator KII 037.***

The other suggestions included the need for specific and clear guidelines specifying the qualifications of the different CAB members. It was reported that this would make it simple to choose community representatives.

*“I think that probably we need to have guidelines, it would be simple of who can be a community representative.” KII* ***REC Chair KII 034.***

“*Policies in form of a framework, guidelines which are very clear how CABs are selected and established and these should be shared with the CAB members.”* ***P021 CAB Chair.***

Empowerment of Research Ethics Committees to regulate CABs was another suggestion given. It was also suggested that Principal investigators should let CABs be independent. This should be monitored by the RECs.

*“I think the RECs should be empowered, we should, to be able to identify where the community Advisory Board is advisable and the way to deal with it, I think it should be left fluid as we get to advance in our research.”* ***REC Chair KII 034.***

*“So, they are set by the PIs, but the PI should not impose things on them. I think that is a key statement. When you go to engage them, you want to find what they think, and what their expectations will be and what your expectations will be. That is but when you start going and you tell them this is what it is, this is what, it becomes a problem, that is where the REC becomes critical to be sure that whatever was planned is going on in the right way. So, the processes of monitoring them becomes critical.”* ***Regulator KII 036.***

Respondents felt that conducting a needs assessment on the intended goal for formation of CABs, their roles, training needs and challenges and the facilitation they need to function smoothly was also suggested before setting up a regulatory framework. They suggested that there should be a focal person at the UNCST to oversee CAB activities and clear guidelines on the formation, the tenure of office, the roles of CABs and qualifications of CAB members and on who facilitates and regulates CABs.

*“It is something someone needs to look through and see where, do like an assessment, what did we want by establishing these CABs and right now, how are they performing and then in the gaps of how they are performing, how often do they meet, which kind of people are on these committees, that kind of assessment and what roles are they doing?”* ***Regulator KII 036.***

*“Because someone the roles may have been too difficult for them and then, that kind of assessment that was done for accreditation of the RECs can also be done, like some kind of assessment of the CABs and then maybe get a particular package, do a training needs assessment and see how evolving even the already existing CAB members and engage them, get their views and see how, you understand their challenges for you to get a model that can work and be sustainable because the other issue with the CABs of course if they are to meet there are these, how would they be met because now like the RECs for them they charge. So, then the running of the CABs unless it is institutional, how then would you hold them to account for their representation.”* ***Regulator KII 036.***

Findings also revealed the need to set up a person at UNCST to oversee CAB formation and activities. It was also recommended that CAB be appointed by the researcher but that REC and UNCST take on the role of approving it.

*“We talked about having a person at UNCST, like the secretariat then the CABs being appointed by the researcher and being approved by the REC and UNCST. And then that particular CAB would work with the regulators, NDA, UNCST, UNHRO, during the implementation. The previous would be reviewing the protocol, development then this one would be during orientation.”* ***Regulator KII 037.***

*“Right now, the community engagement issues are not handled by the UNCST. We just do regulatory oversight by looking into what has already been approved, has researcher A, B and C set up a CAB and then let go but if given, if UNCST decides to for example say okay fine we are looking into CABs definitely they will say that there will be an officer available.”* ***Regulator KII 037.***

It was also recommended that UNCST find ways of generating funds for sustaining different CABs, for facilitation and for running CAB activities.

*“I think the UNCST can find a way of generating funds to sustain the different CABs but also to have a say on what happens and given that it is also in line with the office of the president, they are able to say, district, this person actually belongs to a particular district and is well suited to be there.”* ***Regulator KII 037.***

#### Recommendations on regulation of CAB activities

Respondents observed the need for CABs be to institutionalize so as to remain focused on particular research studies. Additionally, many respondents believed that it’s important for CABs to be adequately regulated by either the REC or the UNCST. Such regulation should be based on clear guidelines on establishment and operations of CABs. It was observed that, independent and regulatory oversight on CABs needs to be informed by clear guidelines. Such guidelines should stipulate who regulates the CABs, what the role of the regulators, the formation of CABs, the composition, the tenure of office, the facilitation and who is responsible for facilitation. There is also need for a person at UNCST to oversee CAB activities.

*“That would be a good option and it would mean we will have to strengthen this technical support supervision and expect the comments directly from the CABs to the regulators. Because, with the regulators there should be a section [in the guidelines] where there is monitoring and evaluation. Because if for instance we are supposed to stop a study, I have to stop it based on this information. So, when the CABs are sharing information, everything must be in writing but they should always give a copy to the regulators.”* **Regulator KII 035.**

*“I would first have to reexamine the role, the current role of the CAB and see where we would fit in because apart from us where we would find that trial needs intervention and then we would engage them, I don’t know whether institutionalization would work for us in terms of role.”* **Regulator KII 036.**

*“I think Community Advisory Boards are going to be there for the individual studies. So, it is an informal structure. It is not a structure that is permanent and the community representative is on the REC so I think, it is enough to monitor how well the communities are taken care of from the activities of the research at the sites. And maybe if we are saying that the number for the community REC could be given out to anybody that might be a CAB but we also know that our chairpersons are representatives of the REC so you just want to de-franchise.”* **REC Chair KII 034.**

The study explored respondents’ opinions on who should regulate CAB activities. And according to study findings it was recommended that UNCST or Research Ethics Committee should do the regulation of CABs to streamline their operations and to promote their independence.

““*Well, UNCST is the regulator. the IRBs are accredited by UNCST so maybe there could be accreditation of CABs by the regulators so that their function is enhanced*.” **KII with Principal Investigator KII 004.**

“*Monitoring CABs should be an active role by the regulators; the regulators should not just sit. And there are many ways they can get their regulation done, either by delegation at the district or by having a formal office with an officer to monitor the CABs activities. And now as they go for monitoring and evaluation now they will be linking up with the community people. So you have a formal function at the same time*.” **Regulator KII 035.**

*“Strengthen the regulatory role at national level which can follow up on what CABs are doing and, work on the M&E framework to use among CABs to assess our work.”* ***P021CAB Chair.***

A minority of respondents felt that there was no need for regulation of CAB activities. This was because there are already many regulatory bodies for research and hence no need for further regulation.

*“I think one of the things that I know, that we are doing now in our research controls, we have several bodies, we have the National Council for Science and Technology, we have the RECs, we have the National Drug Authority. We have the site SO committees or the science committees. Those are enough regulators in my opinion. Let us engage, let us recommend the communities are engaged but let us not create another regulatory layer.”* ***REC Chair KII 034.***

### Training of CABs

Study findings showed that to ensure substantial community engagement by CABs, trial investigators endeavor to prepare CAB members in form of giving them induction briefs on a given clinical trial they plan to undertake. CAB members are basically provided with protocol-specific introductory training that covers the standard operating procedures (SOPs) specific to community engagement for a given clinical trial. The supposed training only includes a lay orientation to the research process and community engagement procedures specific to the trial and this happens at the onset of implementation.

*“We usually give them an overview about the study, target participants, the eligibility and also ask for their input. We also train them on the planned community engagement activities and then we shall need the CABs especially in recruitment and following up participants.* ***KII, Investigator KII 008.***

*“I don’t remember any trainings that we have conducted for our CABs apart from those mini trainings or meetings like the very first CAB meeting where we tell them about their terms of reference, what is expected of them and what is expected from the study team. And then the refresher in the middle so they can always remember their roles* ***KII, Investigator KII 025.***

Other respondents alluded to the fact that CAB members should get trained on other issues important in the conduct of clinical trials such as research literacy and advocacy in community engagement in trials. This however, was often tailored to the interests of the research team in respect of the study they are undertaking. Respondents observed that the training availed to CAB members was inadequate to facilitate appreciation of the research processes.

*“We need them [PIs] to support the CAB. We need more relevant information in trials. As CAB members we need to understand the research language. For example, you are talking about placebo but there are so many members you can ask about placebo and they don’t even know what it is. We need to get to a level where we understand things.”* ***KII, CAB Chairperson KII 011.***

*“No; we don’t have those guidelines apart from the orientation that we get. There is nothing else formal.”* ***P027 CAB Chair.***

However, some CAB chairs felt that the training is either focused on only the research study or non-existent in many situations.

*“No; I don’t think there is any training at all that has occurred and yet that is something that would probably help us build up the standard of our work…” P027 CAB Chair.*

It was noted that to ensure CAB members are adequately empowered to effectively perform their roles, trainings and capacity building for CABs should not be primarily left to the trial investigators and research team. Other stakeholders such as the research regulatory bodies and ethics committees should take up the role of capacity building for CABs to empower them as required.

*“In a training session you may mention one or two ethical issues but spend more time on the project and the integrity. That role shouldn’t be left to the Investigators. Ethical training for the CABs should be a role for the REC.” (****KII, Investigator). KII 005.***

Findings revealed that different research institutions led by principal investigators and the community liaison team determine the training of CAB members both the content, length of the training and period at which the training should be conducted. Such practices do not provide room for standardization of practices across the different studies because the trainings are not guided by any regulations of guidelines.

“Once a CAB is formed, the major bit is training, they need training, they need to be trained, not only on the protocols that we are working on, not only on consent forms but understanding ethics**.” KII with Investigator KII 012.**

*“Usually one day, we have not had it (training) more than a day.”* ***P014 CAB Chair.***

The study revealed that it is the principal investigator that determines the content and length of training for CAB members. According to respondents the content and length of activity varied depending on the type of study and on how fast CAB members comprehended what they were taught.

“*I think it differs from CAB to CAB and it depends on how quickly they understand what you are telling them but there should be training. Understanding the research process, that is critical, and for a given research, they need to understand what it is that the researchers are doing. You know, how are people followed up? What is done during follow up? How the rights and the welfare of participants is kept and so forth, all these things the CAB member should really understand.*” **KII with Principal Investigator KII 004.**

### Challenges regarding regulation of CABs

Challenges of regulating CABs range from lack of clear guidelines, influence by the researcher, the voluntary nature of their work and nature of the study or study requirements. For instance, lack of clear guidelines on the qualification of CAB members or community representativeness of the CAB creates a situation of uncertainty on who should serve as a CAB member.

*“We need to have guidelines; it should be simple to determine the community representativeness of the CAB.”* ***KII REC Chair KII 034.***

Regarding the setup of the CAB, since the CAB is formed by the investigator, regulators cannot advise or question who and why some people are selected to the CAB. This raises issues about quality and standards of CAB activities.

*“These are set up by the researcher so, it is hard to basically say, “can we have so and so included on the CAB.” You can only advice, but you have no legal mandate constituting the CABs.”* **KII Regulator KII 037.**

*“I don’t even know what to say. You see in the meetings somebody writes minutes and reactions are there and at the end of it there is nothing happening. So at the end of it where do we go from there? (Laughs).”* ***P027 CAB Chair.***

*“If they need more power let there be an agent looking out for CABs in Uganda as a whole and there is a framework guiding them to act independently under a certain body at a national level.”* ***P021CAB Chair.***

Independence of CABs and lack of legal mandate of UNCST to constitute the CAB. Some regulators explained that the CABs have some level of independence and that UNCST guidelines do not give regulators the authority to interfere in the work of CABs.

*“These are sort of independent of UNCST and you have no legal mandate constituting the CABs for the researcher. So, it would be good to have different CABs for different clinical trials than having the research institution set up one CAB because each trial has different demands and also has different scientific elements.”* **KII Regulator KII037.**

*“Hmmm that is…you see we are volunteers (laughs) and you see you are limited and you cannot enforce any law. So I think that is where we lie and until that is corrected somehow we will find it very difficult to enforce these recommendations.”* ***P027 CAB Chair.***

The nature of the research study: Different studies present with different requirements. Regulators noted that CABs might be relevant in some studies and not applicable to others.

*“CABs can be very relevant in other projects and in others they are not. For example there are trials that will engage 20 people and these don’t come from a very well-defined community. You won’t have a CAB for them but we still have to make sure that their rights are not abused and to uphold the ethics surrounding the study activities.* **KII REC Chair KII 034.**

#### Volunteer nature of CAB membership

Findings revealed that CABs are voluntary bodies that do not receive salaries but facilitated with sitting allowances during meetings. They are volunteers and when they sit, then they are given transport refund. The study revealed that some institutions budget for CAB activities on a quarterly basis and that funds are drawn from different research project budgets which form a pool from which CAB are facilitated with transport. In some cases, this was highlighted as a challenge that hinders CABs from performing their roles since Principal Investigators or researchers cannot facilitate them.

*“It is really voluntary, they are reimbursed transport, and I don’t know how much they give them like around 50,000 plus, not more than 150,000 shillings.”* ***KII with Principal Investigator KII 033.***

“*Our studies budget for CAB activities, so every study needs to budget for CAB activity because we are going to use them anyway for all our studies, so every single project brings in a small contribution and when they come here, they are reimbursed.”* **KII with Investigator KII 033.**

Respondents observed that RECs review protocols without a salary, and this approach can also be translated to the CABs. Because CAB services are voluntary, it would be possible to sustain a CAB network or committee without salary. However, adequate funding is necessary to facilitate CAB activities.

*“They are volunteers, they are volunteers but now once a CAB is formed, the major bit is training as I have said, they need training. They are given transport refund really because they are just volunteers.”* ***KII Principal Investigator KII 004.***

#### Meetings and feedback mechanisms of CAB members

Study findings revealed that the methods of CABs giving feedback about their activities and issues in their communities varied across different research institutions. Some of the methods reported included free discussions, use of email, phone calls, sharing during the Cross-CAB Network and walk ins in case something is very urgent.

*“I don’t have like real specific times when they meet but I think generally there is free discussions if they have heard any myths and misconceptions, they can call the community team, the team members directly, they can send emails, they can meet at meetings and then there is the annual cross CAB Network that happens in November.”* ***KII Principal Investigator KII 033.***

*“If there is anything urgent, a CAB member can come to the site, if things may not be urgent, that can be discussed during the meeting.”* ***KII Investigator KII 004.***

Findings revealed that investigators are responsible for organizing CAB meetings and determine the agenda of the day. However, sometimes such meetings do not occur or take long to happen.

*“We normally meet once a quarter and when they come here, we give them an overview of how things are going, we listen to them on how, the last time we spoke about that what happened and even new studies, we introduce them to new studies and get their views on new studies, so there is usually a clinician that presents to them, every CAB meeting there must be a clinician and then the community team and we all sit together and discuss.”* ***KII Investigator KII 033.***

*“Yah but sometimes the resources to mobilize people are not there which is understandable. I always look for opportunities from my constituency where I can talk to people which are already paid for and are not initiated by me. So, I pass on the message and interact with people only if there is another activity going on.”* ***P 021 CAB Chair.***

#### Independence of CABs

It was observed that the independence of CABs is questionable since they are formed by investigators that are responsible for the concerned research study. Some respondents noted that independence was necessary but there has to be some form of regulation.

*“The issue of independence is one that is challenging, but I think what would make them a little more independent is the fact that they are not chosen by the researchers. If they were chosen by researchers and paid a stipend by researchers, I think that they would not be independent because I look at the salary you pay and say but given that they are volunteers, we don’t pay them and we don’t choose them I suppose that there is some degree of independence.”* ***KII Investigator KII 004.***

*“Yeah; the CAB needs to be empowered. For instance we have not met and it is now coming to a year.”* ***P027 CAB Chair.***

Some few respondents felt that this idea of independence was just theoretical but the reality was different.

*“Theoretically, they are independent but practically they are not, even us as investigators we are not entirely independent. For we do this work on behalf of someone. Further, we as PIs have interests concerning the studies to protect, so even if CABs want to operate independently our decisions would override their recommendations.”* ***KII with Investigator KII 001.***

Some respondents proposed that for CABs to be independent, principal investigators should not impose things on them. It was suggested that expectations from both CABs and Principal investigators should be spelt out at the start. Hence the need for a regulatory agency to oversee and ensure that CABs remain independent and not influenced by researchers.

*“The independence would come in if there is an authority that first of all has a legal mandate and also independent of research because UNCST apparently does not carry out clinical trials or research, so, if UNCST is able to look into the different CABs that are being formed and actually have a say on what is going on.”* ***Regulator KII 037.***

Respondents observed that for CABs to be independent, and to make decisions independently the legal mandate to regulate CABs has to be given to an independent body rather than being left to principal investigators. However, this approach has a challenge of striking the balance of facilitating the CAB members and also making sure that they stay objective and they are independent and they are doing their work in the best interest of the communities they represent.

*“We are banking on the fact that people (PIs) have the highest honesty. Otherwise, really there can be a lot of compromise. But I also see there is a potential conflict many times when those community-based people have not been given money because they are not part of the program. They think it is an allowance*. **Regulator KII 035.**

*“This is very difficult…because you may say okay, I am appointing one person who is answerable to me. But now here, you don’t know where the PI is going to come from. So that one we have to think about it, how you can involve the community without compromising it. Because they will not come if you do not pay them; you have to disguise the payment maybe as transport or something like that. But don’t give them a salary; just give them a small token and say this is your transport*. **Regulator KII 035.**

*“There is need for institutionalizing them and attaching them to local authorities so that they are not necessarily linked to the PI, because they are the ones to pay them*- **Regulator KII 036.**

## Discussion

We assessed existing regulatory and monitoring mechanisms for CABs in Uganda and our findings revealed that the CABs are appointed by the research institution/researcher, operate under the guidance of the researcher and lack guidelines or regulatory oversight by the UNCST and or RECs. Respondents felt that CAB operations and activities should be regulated by the national regulators or RECs with a hope that this would streamline operations and promote independence. They felt that regulators should determine the kind of guidelines or manuals to standardize operations of CAB activities at the different research institutions.

The absence of adequate guidelines and mechanisms for proper regulation of CABs is a recipe for loopholes and different standards of operation. Regulation of CABs is necessary in promoting transparence, accountability and improved ethical conduct of research [16, 28, 30]. This can also help regulators appreciate the nature and extent of community engagement carried out by the CABs.

Guidance for CABs and other related community engagement approaches has been provided by some initiatives [33]. However, only well guided and regulated initiatives can perform their functions effectively [33]. Such initiatives used generic, non-contextualized guidelines developed for HIV prevention trials. CAB Charters have also been used to guide CABs though not informed by country specific guidelines. The data generated from this study can inform contextualized guidelines for regulation of CABs and facilitate standardization in the of community engagement process. This is expected to contribute to improved ethical conduct of research in Uganda.

Respondents further highlighted the fact that since the RECs review protocols, monitor implementation of research activities and ensure dissemination of research findings, they can as well oversee CAB activities. This is in line with the fact that the community engagement has been mandated for almost all clinical trials and is increasingly becoming an integral component of the research process as highlighted by scholars in South Africa [34]. Regulation of the CABs by the concerned REC or national regulator can facilitate adequate oversight on the community engagement aspects of the research. This is in line with research ethics regulations and guidelines in Uganda that require RECs to provide oversight for most of the research related activities [3].

A number of challenges affect the current operation of the CABs including CABs being appointed and working for the researcher, the lack of clear guidelines, the voluntary nature of their work and, limited training. Additionally, CABs rick loosing Independent because their activities are facilitated and sorely depend on the investigator whose research, they facilitate community engagement. This challenge is similar to that faced by RECs which are established and funded by the host institution, yet their functions are expected to be independent. Thus, if RECs can perform their functions well, so does a well constituted CAB provided guidelines and regulatory oversight are adequate. Although CABs may not be fully independent, with proper guidelines and regulation, they are expected to meet some minimum standard.

Additionally, the lack of adequate and sustained funding for CAB activities coupled with inadequate training and skills to perform their roles may have negative implications on ethical conduct of research. These challenges can be addressed through a number of mechanisms including; regulation by UNCST and RECs, development of guidelines for effective community engagement and CAB activities, mechanisms for funding CAB activities that are independent of the investigator a well as regular and appropriate training. These proposed mechanisms are feasible based on the fact that even the RECs tend to operate under similar circumstances. These challenges of CABs in Uganda are not unique as literature highlights a number of issues including incomplete ethical regulations and guidance; limited knowledge of science among members of communities and CABs; unstable and unbalanced power relationships between researchers and local communities; poor CAB management, including lack of formal participation structures and absence of CAB leadership; competing demands for time that limited participation in CAB activities; and language barriers between research staff and community members as well as shortcomings within the research team [35, 36].

Although different categories of stakeholders were studied, their views on CAB activities, challenges and proposed mitigation plans are quite similar. The stakeholders to a great extent are in agreement concerning the need for regulatory oversight for CABs, development of guidelines for CAB establishment and operations, training in research ethics related aspects and some level of independence. This highlights the necessity for ethical regulation and oversight for community engagement in research. And if such regulation is effectively carried out, it can lead to improved community engagement with resultant ethical conduct of research.

Other considerations for guideline development and regulation of CABs include the need for trial specific CABs. This would enable a CAB to concentrate on a specific research protocol and concerned community for improved functionality rather than working on all the research projects conducted at the institution. Other aspects that could improve CAB functionality include setting minimum qualifications for CAB members, conducting a needs assessment of CABs as well as adequate and continued training on the protocol and research ethics. Such capacity improvement is essential for better quality performance [35, 36].

## Conclusion

The study shows that CABs exist in Uganda and their formation is guided by a section in the national guidelines for conduct of research involving humans as participants which is not adequate guidance. Respondents highlight the need for clear and adequate regulation and oversight of CABs by regulatory bodies based on detailed guidelines and frameworks. The functionality of CABs is however hindered by several challenges which including lack of knowledge and training in research ethics, lack of resources to facilitate their work, lack of independence, and lack of guidelines for their operations.

## Supplementary Information

**Additional file 1.** Interview guide.

## Data Availability

Data sources are available on request. Request can be made to the corresponding author at ochiengjoe@yahoo.com

## Abbreviations

CAB: Community Advisory Board
KII: Key Informant Interviews
REC: Research Ethics committee
CAB: Uganda National Council for Science and Technology
