# Supplementary material for: Regulation of community advisory boards during conduct of clinical trials in Uganda: a qualitative study involving stakeholders
Source: BMC Health Serv Res. 2023 Feb 6;23:119. doi: 10.1186/s12913-023-09136-w (PMC9899660; doi:10.1186/s12913-023-09136-w)
Supplement: Supplementary file 1 — Additional file 1. Interview guide. [file 12913_2023_9136_MOESM1_ESM.docx]

# Appendix 1: Data collection tools

**Key Informant Interview Guide for Regulatory bodies.** *Target audience: UNCST, NDA, UNHRO, RECs*

**Brief introduction of the study**

***We are collecting data on past and existing community engagement structures in clinical trials. In particular, we aim to assess the existence and functionality of Community Advisory Boards (CABS) and conduct a needs assessment to identify capacity and training gaps of the CABs. The information you provide will inform the development of guidelines and training manual for CABs in the country.***

**Some background questions**

1. Age
2. Sex
3. Place of work/ institution
4. Occupation
5. Field of specialization
6. Highest level of education attained
7. Duration of service as a research regulator
8. In your opinion, what do you understand by community engagement?
9. What community engagement structures are available in Uganda for clinical trials? *Prompts: CABs, patient advocacy groups, community advisory groups, local health service providers, stakeholder groups, community advocates and opinion leaders, and mass media (Tv, radio talk show, newspapers, adverts and posters)*
10. What is the role of community leaders in clinical trials? Do they have capacity to perform their expected roles?
11. The Ethics guidelines of 2014 recommend that PIs form CABs for their clinical trials. As a regulator could you tell us about the situation of CABs in Uganda? [Probe into: Number of CABs in the country, guidelines for CABs formation, appointment to the CAB, membership requirements, role of CABs, tenure of office, training, experience in community engagement and ethics, knowledge of the local context, etc
12. What is your opinion about creating a formal link between CABs and the regulator? What would be your role in this link? And the role of the CABS in this link?
13. In your opinion, how should Investigators ensure that CAB members are competent to perform their functions? Probe into: trainings and refresher courses for members in ethics and research conduct, commitment to the CAB, involving the members in decision making, being available to support the members.
14. Any other information you would like to share with us?

Thank you.

**Key informant guide: REC Members/Chairpersons**

**Brief introduction of the study**

***We are collecting data on past and on existing community engagement structures in clinical trials. In particular, we aim to assess the existence and functionality of Community Advisory Boards (CABs) and conduct a needs assessment to identify capacity and training gaps of the CABs. The information you provide will inform the development of guidelines and training manual for CABs in the country.***

**Some background questions**

1. Age
2. Sex
3. Place of work/ institution
4. Occupation
5. Field of specialization
6. Highest level of education attained
7. Duration of service as REC member

**Guiding questions**

1. In your opinion, what do you understand by community engagement in clinical trials?
2. How does the REC ensure that community engagement activities have been adhered to:

a) Prior to protocol approval

b) During protocol implementation

c) At the closure and post-trial activities

1. What is your opinion about soliciting the input of CABs into the design, conduct and post-trial activities? Do they matter?
2. Would you consider community engagement as a key criteria in REC determinations *(approval, disapproval or require for modifications of clinical trial protocols*), and why?
3. Does your REC have a standard for scoring community engagement approaches? **Yes or No** If yes please take us through the scoring criteria. If No what does your REC base on to approve that a protocol meets the minimum requirements for community engagement?
4. Do you know of any national or international guidelines for community engagement could you please mention or elaborate on them? Are the National guidelines for research sufficient in addressing community engagement?
5. Do you think adopting the international guidelines for CABs will serve the local context of Uganda?
6. What is the role of a community representative on your REC?
7. Evaluate/Assess the contribution of the community representative to the REC. Are they relevant on the committee? *Do they address the views of the community?*
8. In your opinion is it necessary to create a link between the community representatives on the REC and CABs? And how do you think this can be done?
9. Have you attended any training in research ethics with particular emphasis on community engagement in clinical trials?
10. Community advisory board is an idea that was initiated in the western countries. We would like to get your opinion on whether this applies to the Ugandan local setting and if it serves its intended purpose. Do you think that the name CAB suits its role in the local context?
11. Currently CABs are established by PIs, in your opinion what mechanisms do you recommend for regulating CABs?
12. What is your opinion on the independence of the CABs to take decisions during a clinical trial
13. Do you have any other information you would like to share with us?

Thank you.

**In depth Interview: CAB members/Chairpersons**

Brief introduction of the study

We are collecting data on past and on existing community engagement structures in clinical trials. In particular, we aim to assess the existence and functionality of Community Advisory Boards (CABs) and conduct a needs assessment to identify capacity and training gaps of the CABs. The information you provide will inform the development of guidelines and training manual for CABs in the country.

Some background questions

i. Age

ii. Sex

iii. Place of work/ institution

iv. Occupation

v. Field of specialization

vi. Highest level of education attained

vii. Duration of service as a CAB member

Guiding questions

1 In your opinion, what do you understand by community engagement in clinical trials?

2 Describe the methods you have used to engage with the communities to participate in research.

3 From your experience as a CAB member, what reasons do participants give for their participation and non-participation in clinical trials?

4 What is your role as a CAB member/Chairperson?

5. How did you become a CAB member? Are there any guidelines/ procedures/SOPs that define membership? Please explain.

6. As a CAB member, explain how your activities are monitored and regulated? By who, when, for what purpose, etc.

7. Have you attended any trainings/ refresher courses in research ethics and human rights protection in clinical trials? Probe into: types of trainings, duration, certification, frequency, who conducted the training, etc.

8. Have you ever raised any recommendations from the community to the investigator? How was it addressed?

9. Community advisory board is an idea that was initiated in the western countries. We would like to get your opinion on whether this applies to the Ugandan local setting and if it serves its intended purpose. Do you think that the name CAB suits its role in the local context? Should it change? Please propose

10. Currently CABs are established by PIs, in your opinion what mechanisms do you recommend for regulating CABs?

11. What is your opinion on the independence of the CABs to take decisions during a clinical trial

12. How have you performed your activities as a CAB member? Probe; community entry, community education, consultations with the communities, consultation with the investigators, advising community on pertinent issues, etc.

13. In your opinion as a CAB member, do you feel that your views in regards to community are considered by the PI?

14. How do you assess your role as a CAB member? (no of meetings, contributions to society, recognition by UNCST, PI or the society, merit , invitation for tr

**Key Informant guide: Trial Investigators/Community Liaison Officers**

**Title of the study:**

**Some background questions**

1. Age
2. Sex
3. Place of work/institution
4. Occupation
5. Field of specialization
6. Highest level of education attained
7. Duration of service as investigator

**Brief introduction of the study**

***We are collecting data on past and on existing community engagement structures in clinical trials. In particular, we aim to assess the existence and functionality of Community Advisory Boards (CABS) and conduct a needs assessment to identify capacity and training gaps of the CABS. The information you provide will inform the development of guidelines and training manual for CABs in the country.***

**Guiding Questions**

1. What do you understand by community engagement in clinical trials?
2. What are the different types of community engagement approaches you know?
3. Share with us the different community engagement approaches you have used for the different trials you have been involved in and your experience working with them. ( probe for aspects of establishment, facilitation , functionality and feedback mechanisms
4. Which community engagement approaches have worked and why? Which approaches didn’t work and why?
5. Community advisory boards are one of the community engagement approaches. What is your opinion about their functionality and effectiveness?
6. What’s your opinion about CAB’s knowledge and understanding of:

Prompts

- local cultures and perspectives.
- languages,
- dynamics of local trials,
- concerns of vulnerable or marginalized populations, and
- local priorities.

1. How are the CABs established or formed? Are there any guidelines or procedures***?*** Are these guidelines followed as reference documents during the formation of the CABs (examples of Guidelines: GPP for Biomedical Trials, 2011, *Prompts: appointments, membership requirements, SOPs, guidelines, manuals*
2. How are CAB members identified?
3. How are CAB members selected? How are their roles and tenure of office decided?

Probe: *Representation, number, cultural insight, technical expertise, access and leadership potential, commitment to advance research, voluntarism, maintaining confidentiality and commitment to the CAB’s mission, roles and responsibilities.*

1. How do Investigators ensure that CAB members perform their functions? Probe into: *trainings and refreshers courses for members in ethics and research conduct, commitment to the CAB, involving the members in decision making, being available to support the members,*
2. Have you conducted any training for your CAB? If yes please elaborate on the types or fields of the training, frequency, duration and mode of delivery.

How are the CAB activities monitored and evaluated?

1. How often do you receive feedback from your CAB? What type of recommendations have you received (closure, amendment, termination etc…) and how do you act on their recommendations? How do you act on issues of violation of participant’s rights and non-compliance?
2. Who regulates CABs activities? Prompts: *regulator, investigator, research institution, Monitoring, reporting, meetings, etc.*
3. What challenges do CABs face in performing their roles? What can be done to solve the challenges?
4. Community advisory board is an idea that was initiated in the western countries. We would like to get your opinion on whether this applies to the Ugandan local setting and if it serves its intended purpose. Do you think that the name CAB suits its role in the local context?
5. Currently CABs are established by PIs. In your opinion, what mechanisms do you recommend for regulating CABs?
6. Any other information you would like to share with us?

Thank you.
